# Anterior-Only Sacrocolpopexy Complicated by Obstructive Enterocele: Should Both Compartments Always Be Repaired?

**DOI:** 10.7759/cureus.99038

**Published:** 2025-12-12

**Authors:** Leandro Nobrega, Boris Schiltz, Jerome Mathis, Caroline Eggemann

**Affiliations:** 1 Department of Obstetrics and Gynecology, Spitalzentrum Biel, Biel, CHE; 2 Department of General Surgery, Spitalzentrum Biel, Biel, CHE

**Keywords:** mesh repair, obstructive enterocele, pelvic organ prolapse, rectocele, sacrocolpopexy

## Abstract

Sacrocolpopexy is widely regarded as the gold standard for apical prolapse repair, yet the extent of mesh placement remains debated. While some surgeons routinely place both anterior and posterior meshes, others favor a compartment-specific approach. We report the case of a 60-year-old woman with prior hysterectomy and anti-incontinence mesh surgery who underwent anterior-only robot-assisted sacrocolpopexy for apical and anterior prolapse. Six weeks later, she developed incomplete bowel emptying, pelvic pressure, and a posterior vaginal bulge. Examination revealed posterior compartment prolapse, and MRI defecography confirmed an obstructive enterocele with small bowel descent, rectocele, and intussusception. After multidisciplinary discussion, she underwent robot-assisted posterior sacrocolpopexy combined with rectopexy, including posterior mesh placement and Douglas closure. The postoperative course was uneventful, and symptoms resolved at the four-month follow-up. This case highlights obstructive enterocele as a rare but clinically significant complication of anterior-only sacrocolpopexy and underscores the need for individualized surgical planning and vigilance for postoperative bowel dysfunction, supporting a selective approach in which posterior support may be considered when posterior compartment weakness or bowel dysfunction is evident.

## Introduction

Pelvic organ prolapse (POP) is a prevalent condition worldwide, with symptomatic rates ranging from 3% to 10% depending on population and diagnostic criteria [1]. Its impact extends beyond anatomical changes, as women often experience pelvic pressure, urinary and bowel dysfunction, and sexual difficulties, all of which impair quality of life [2,3]. The burden of POP is expected to rise with global population ageing, even though age-standardized rates are stable [1]. These epidemiological and functional considerations reinforce the clinical relevance of compartment-specific assessment, since pelvic dysfunction may differ according to anterior and posterior involvement.

Surgical correction remains the mainstay of treatment for advanced or symptomatic cases. Among available techniques, sacrocolpopexy - performed by open, laparoscopic, or robotic approaches - is widely regarded as the gold standard for apical repair, owing to superior long-term anatomical and functional outcomes [4,5]. Vaginal suspensions, such as uterosacral and sacrospinous ligament fixation, remain relevant alternatives, particularly for frail or older patients [6,7].

Within sacrocolpopexy, the extent of mesh placement remains debated. Some surgeons advocate routine use of both anterior and posterior meshes, while others adopt a compartment-specific strategy. In several European centers, it is common to perform anterior-only sacrocolpopexy when no posterior defect is present [8,9]. The rationale includes shorter operative time, lower morbidity, and avoidance of posterior mesh-related complications such as constipation, pain, and mesh exposure [8-11]. Reported posterior recurrence rates after anterior-only repair range from approximately 17% to 31% in the absence of a pre-existing posterior defect, although current evidence is limited to small retrospective cohorts, and randomized trials are lacking [8,9].

Despite these considerations, posterior compartment dysfunction remains clinically significant. Although uncommon (<1% in published sacrocolpopexy series), bowel-related complications, including obstructive symptoms, can arise after isolated anterior repair, sometimes in the early postoperative course, leading to severe symptoms and occasionally requiring reoperation [12-14]. Whether prophylactic posterior support should be added in all apical repairs remains an unresolved question, as reflected in current international guidance, and further evidence is needed.

We report a case of symptomatic obstructive enterocele occurring shortly after anterior-only sacrocolpopexy, confirmed by defecography and requiring reoperation with posterior mesh placement. This case illustrates a potential complication that warrants careful consideration in surgical planning and contributes to the ongoing discussion about whether both compartments should be addressed during apical prolapse repair.

## Case presentation

A 60-year-old woman with a history of hysterectomy and prior tension-free vaginal tape (TVT) procedure more than 20 years earlier was referred to our urogynecology department in May 2025 for persistent defecatory dysfunction and vaginal bulging after a recent prolapse repair. She was a non-smoker, had a BMI of 33.1 kg/m², and no clinically relevant comorbidities or chronic medications that might impair wound healing or surgical outcomes.

In January 2025, she had undergone a robot-assisted sacrocolpopexy at another institution for apical and anterior compartment prolapse. During this procedure, only an anterior mesh was implanted, anchored at the vaginal apex, and fixed to the promontory. Pre-operative clinical assessment before the index procedure demonstrated a stable posterior compartment without symptomatic rectocele or enterocele, and no bowel-related complaints were reported. The early postoperative course was unremarkable. However, within six weeks, she reported new pelvic pressure, incomplete bowel emptying, and a posterior vaginal bulge. As a conservative measure, a cube pessary was initiated to provide temporary posterior compartment support and to evaluate whether symptom improvement could be achieved without early reoperation. Conservative bowel regulation was also attempted. However, despite pessary use and dietary measures, her symptoms persisted, and she sought further evaluation.

**Figure 1:**
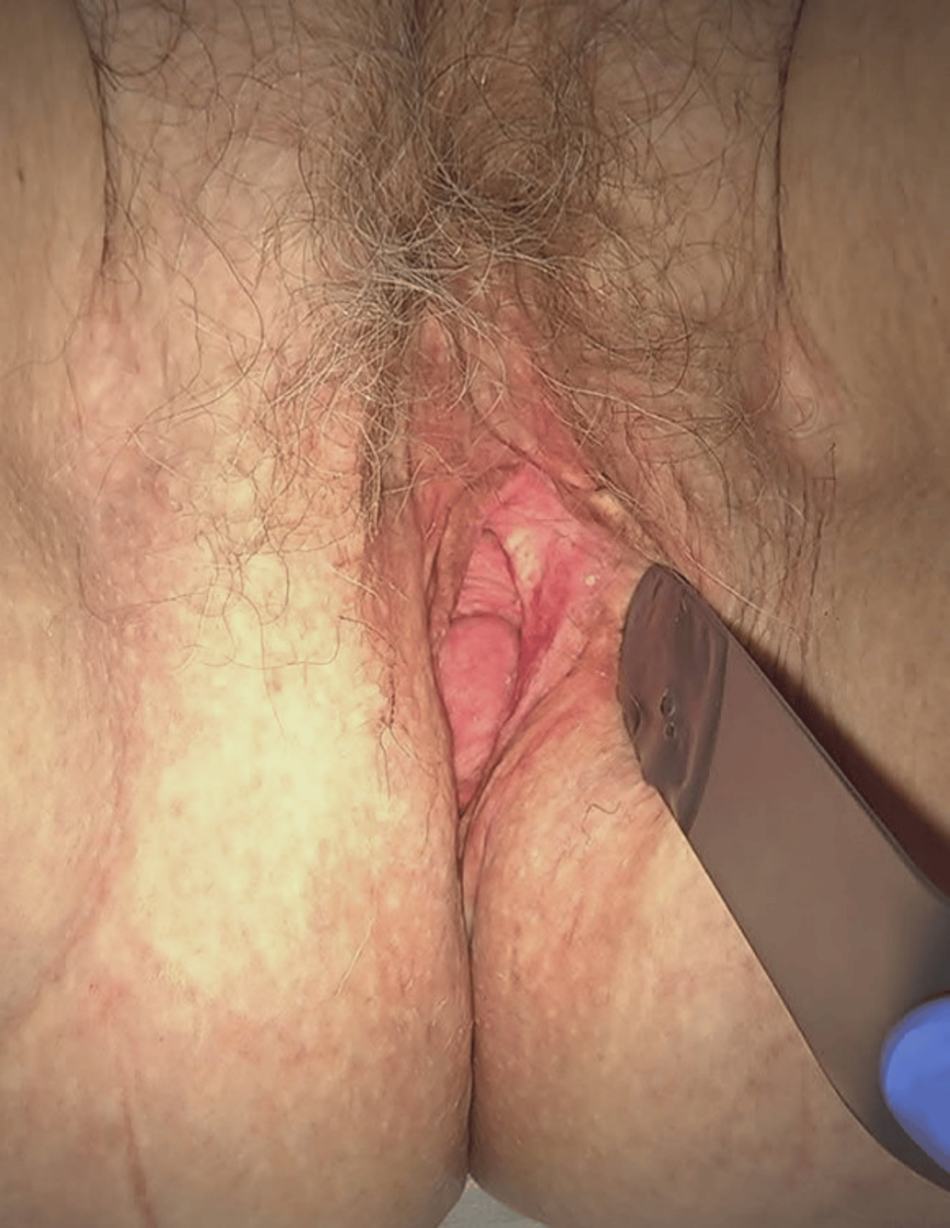
Clinical image demonstrating posterior vaginal wall prolapse/enterocele at presentation. The posterior compartment bulges toward the introitus under gentle retraction, consistent with symptomatic posterior descent.

On examination, the anterior compartment was well supported by the previously placed mesh, and the vaginal apex was stable. A complete Pelvic Organ Prolapse Quantification (POP-Q) assessment demonstrated Aa -2 cm, Ba -2 cm, C -6 cm, Ap +1 cm, Bp +1 cm, GH 3.5 cm, PB 3 cm, and TVL 8 cm, corresponding to a posterior compartment POP-Q Stage II (Figure 1). Combined vaginal and rectal examination suggested a rectocele and an enterocele. The patient also reported occasional stress urinary incontinence with heavy physical activity but denied urgency or nocturia. A validated questionnaire, the International Consultation on Incontinence Questionnaire-Urinary Incontinence Short Form (ICIQ-UI SF), revealed mild stress incontinence, not significantly impacting daily life.

**Figure 2:**
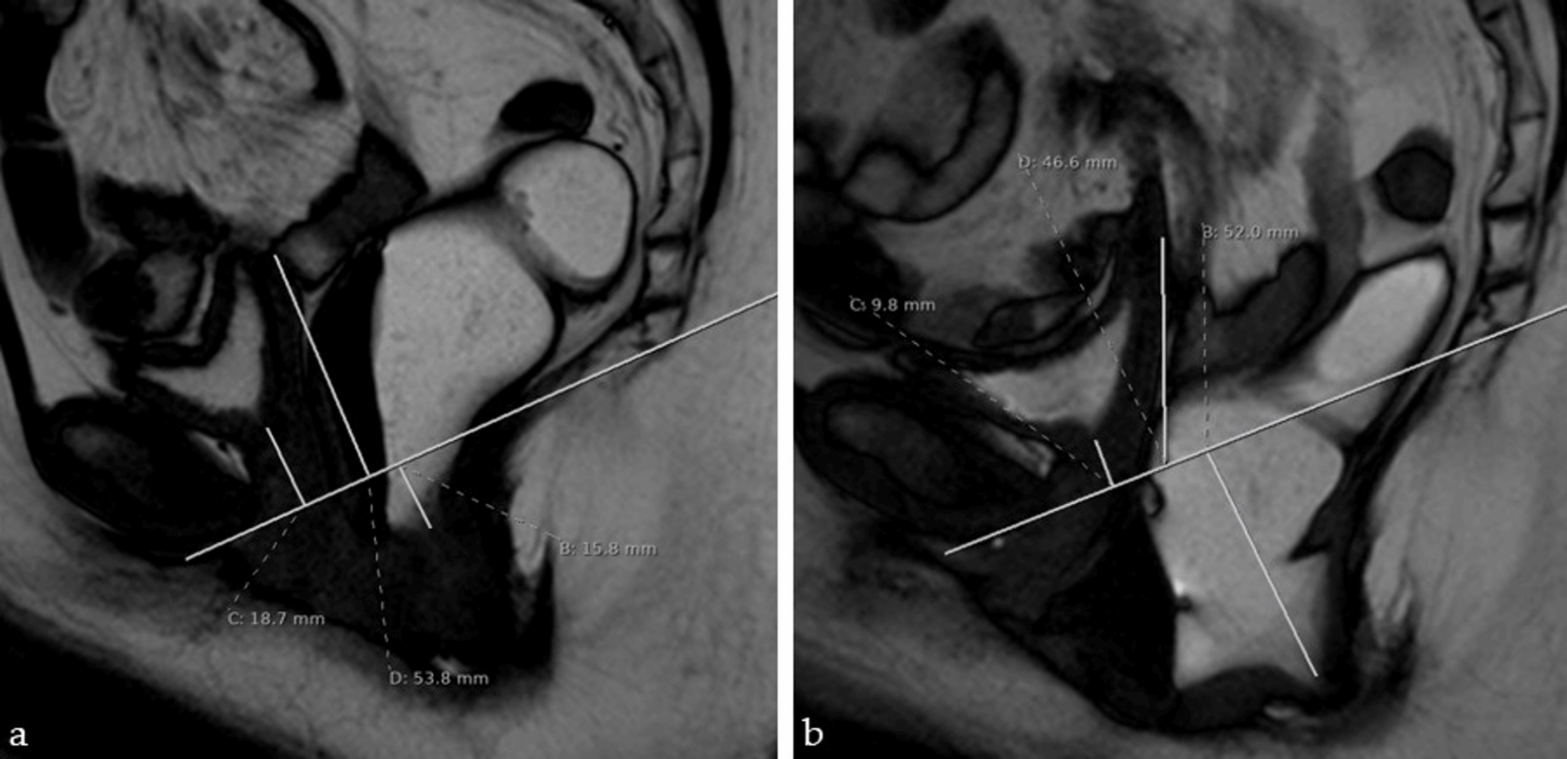
MRI defecography, sagittal view. (a) At rest, anterior, middle, and posterior compartment descent measured relative to the pubococcygeal line (PCL): 1.9 cm, 3.4 cm, and 1.6 cm, respectively, demonstrating mild posterior descent. (b) During evacuation, descent below the PCL increases to 1.0 cm (anterior), 4.7 cm (middle), and 5.2 cm (posterior), revealing marked posterior compartment collapse with rectocele formation and an obstructive enterocele measuring 52.0 mm.

MRI defecography demonstrated a moderately severe enterocele with a small anterior rectocele, mild intrarectal intussusception, and obstructive descent of the small bowel into the rectovaginal space (Figure 2a, 2b). As no universally accepted radiological grading system exists for enterocele severity on dynamic MRI, severity was described based on the extent of small-bowel descent below the pubococcygeal line. These findings confirmed the suspicion of an obstructive enterocele explaining the patient’s defecatory dysfunction. 

The case was discussed in an interdisciplinary pelvic floor board with colorectal surgery. Given the obstructive enterocele and symptomatic recurrence, surgical re-intervention was recommended. Before deciding, the patient received multidisciplinary counseling, including discussion of non-surgical options such as pelvic floor physical therapy, bowel management strategies, and supportive measures. However, given persistent obstructive symptoms and limited expected benefit from conservative management, the patient elected to proceed with surgical treatment after shared decision-making.

**Figure 3:**
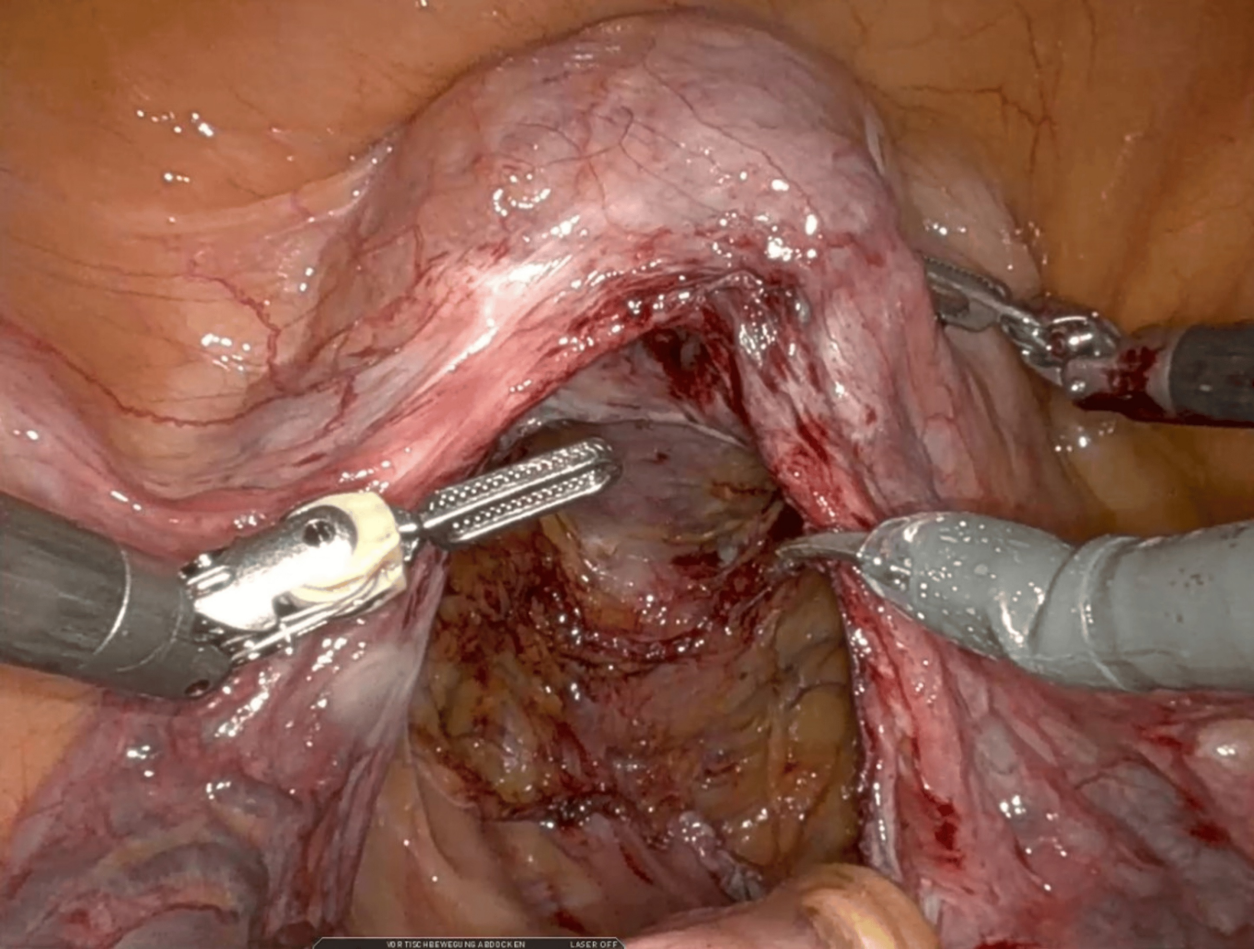
Initial intraoperative view with the posterior vaginal wall and Douglas pouch mobilized.

**Figure 4:**
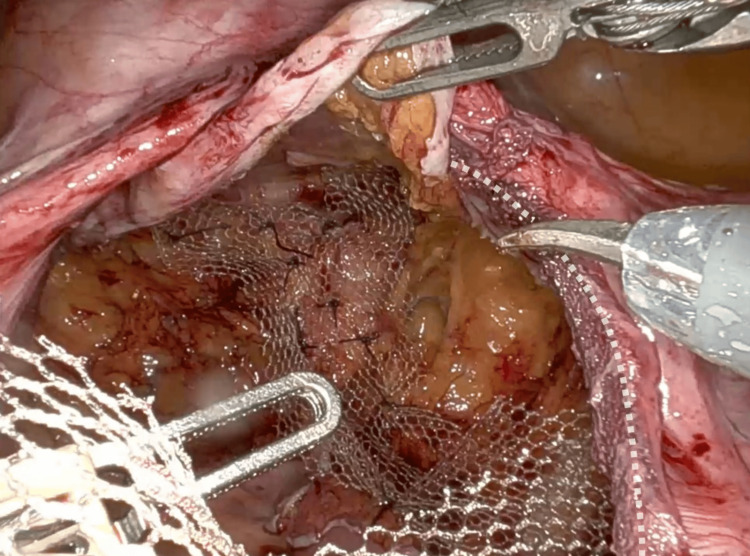
Mesh placement with fixation at the rectum. Dashed line: well-positioned anterior mesh fixed at the sacral promontory.

**Figure 5:**
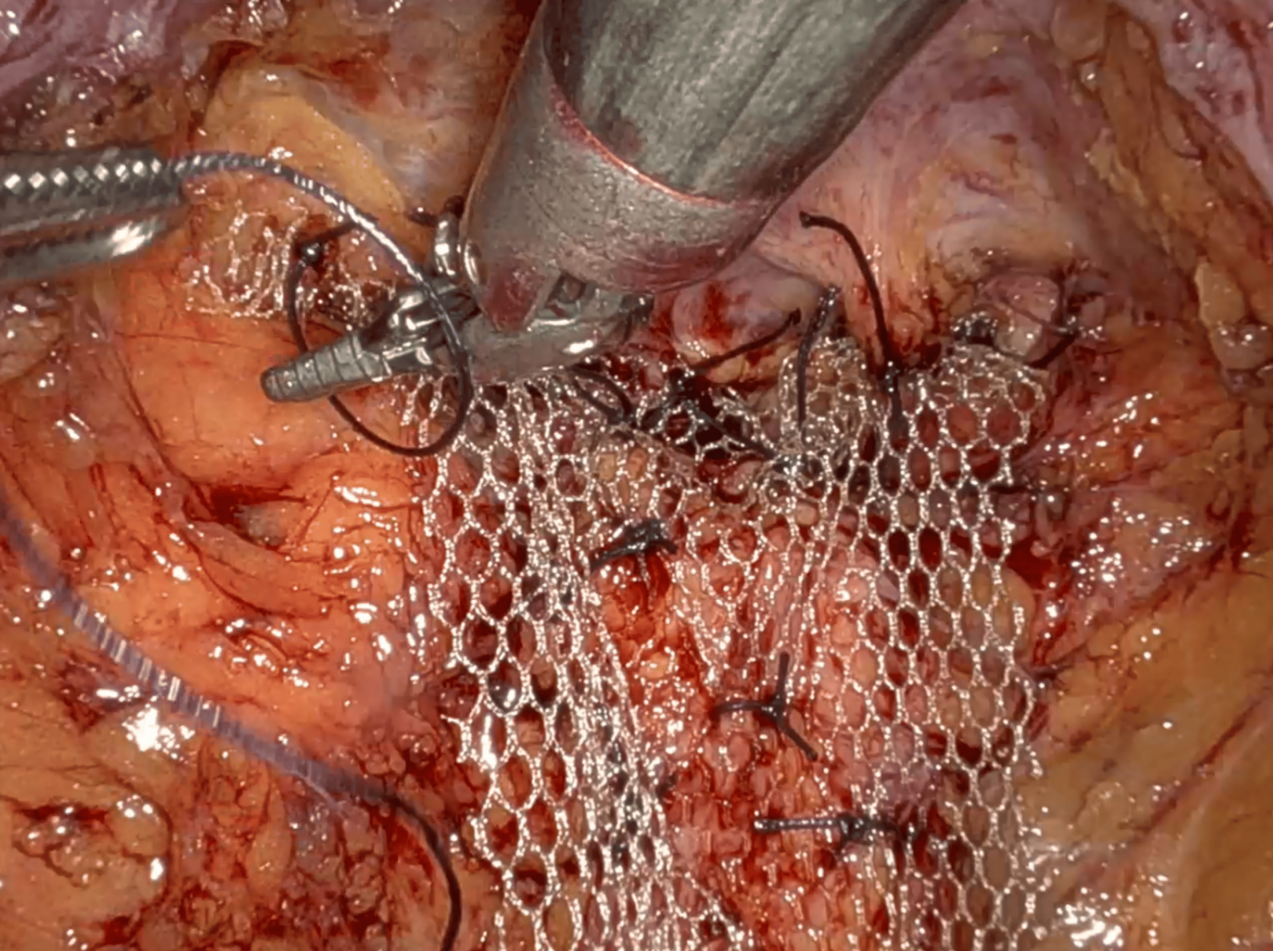
Closure of the Douglas pouch with mesh fixation, already fixed at the rectum and anchored to the vagina.

In August 2025, she underwent robot-assisted posterior sacrocolpopexy combined with rectopexy (Figure 3). A posterior lightweight macroporous monofilament polypropylene mesh (PelviGYNious®) was trimmed intraoperatively to approximately 8 × 15 cm, covering the rectovaginal defect, fixed laterally to the posterior vaginal wall, and anchored to the sacral promontory (Figure 4). The pouch of Douglas was closed, and the new mesh was securely integrated with the pre-existing anterior mesh placed during the index surgery, which consisted of a monofilament PVDF implant (DynaMesh®-PRS Visible) designed for apical sacrocolpopexy. The rectum was simultaneously suspended (rectopexy) to correct the associated intussusception (Figure 5). The procedure lasted 133 minutes (console time 107 minutes), with minimal blood loss and no intraoperative complications.

The postoperative course was uneventful. The patient was managed according to the institution’s enhanced recovery after surgery (ERAS) protocol, which included early mobilization, early oral intake, multimodal, opioid-sparing analgesia, and standardized bowel regulation. She was discharged on the third postoperative day in good general condition, continent, and with pain well controlled by non-opioid analgesics.

At the four-month follow-up, she reported marked improvement in pelvic pressure and bowel emptying, with no recurrence of bulging or obstructive symptoms.

## Discussion

Sacrocolpopexy is considered the gold standard for the management of apical prolapse, with long-term anatomical and functional success rates superior to those of native tissue vaginal repairs [4,5]. Despite this, important controversies remain regarding the extent of mesh placement, particularly whether both anterior and posterior compartments should be routinely supported. In many European centers, anterior-only sacrocolpopexy is commonly performed when no posterior defect is observed, with the rationale of reducing operative time, limiting morbidity, and avoiding mesh-related posterior complications such as constipation, pain, and dyspareunia [8-11].

The present case illustrates that this strategy may rarely be complicated by symptomatic posterior recurrence, in this instance, an obstructive enterocele requiring surgical re-intervention. Importantly, no rectocele or posterior compartment defect was documented preoperatively, reinforcing that the obstructive enterocele represented a de novo postoperative dysfunction rather than progression of pre-existing pathology. Reported rates of de novo posterior prolapse after anterior-only sacrocolpopexy range from 10% to 29%, a variability likely attributable to differences in definitions of posterior failure, follow-up duration, and patient characteristics across published series [8-12]. The need for reoperation is substantially lower, often below 5% [8-12]. Pacquée et al. reported posterior anatomical failure in 28.6% of patients after laparoscopic sacrocolpopexy, although most recurrences were asymptomatic and rarely required reoperation [12]. Thus, while posterior failure is not uncommon radiologically, clinically significant symptomatic enterocele remains an unusual outcome.

Comparative studies assessing anterior-only versus combined anterior and posterior mesh placement suggest that a compartment-specific approach is safe and effective. Rusavy et al. reported that single-compartment mesh placement achieved outcomes comparable to dual-compartment mesh in terms of recurrence and functional results, with the advantages of shorter operative time and hospitalization [8]. Likewise, Antiphon et al. argued that posterior mesh placement is not mandatory in the absence of a rectocele or posterior defect, as systematic posterior augmentation increased posterior compartment complications without improving results [9]. Recent randomized data also raise concerns: Di Biasi et al. demonstrated that posterior mesh fixation increased the risk of postoperative constipation compared with non-fixation (13% vs. 3.3% at one year), supporting the view that routine posterior repair may impair bowel function [10].

Importantly, the most recent Cochrane Review on apical prolapse surgery [4] describes sacrocolpopexy as typically involving both anterior and posterior mesh placement, but found no randomized trials directly comparing anterior-only versus combined approaches. Functional outcomes, particularly bowel symptoms, were inconsistently reported, and the available evidence was insufficient to determine whether systematic posterior mesh placement provides additional benefit [4]. This highlights the persisting knowledge gap and supports the need for further prospective studies specifically addressing compartment-specific strategies.

Functional outcomes after sacrocolpopexy highlight the complexity of the relationship between anatomy and symptoms. Several studies confirm that bowel function often improves after apical suspension, regardless of whether posterior repair is added [15-17]. In a prospective series, Bradley et al. found significant improvement in obstructive and irritative bowel symptoms one year postoperatively, with no clear advantage to concomitant posterior repair [16]. Similarly, Crane et al. demonstrated that de novo outlet constipation occurred at low rates and was not significantly influenced by posterior repair [17]. These findings suggest that while posterior mesh can enhance anatomical correction, it does not consistently translate into better bowel outcomes.

On the other hand, persistent or de novo obstructed defecation after sacrocolpopexy has been reported in up to 10-20% of women [15,18]. Baessler and Schuessler described altered defecation in 28% of patients after abdominal sacrocolpopexy, potentially related to surgical denervation or altered rectal anatomy [18]. Furthermore, Pacquée et al. noted that 11.8% of women who felt worse after laparoscopic sacrocolpopexy cited obstructive defecation as the main reason, even in the absence of recurrent prolapse [12]. Taken together, these findings highlight that functional bowel symptoms may persist or arise postoperatively, independent of anatomic success, emphasizing the multifactorial etiology of obstructed defecation.

Mechanistically, apical fixation directed predominantly to the anterior compartment may modify pelvic force vectors and reduce posterior fascial tension. When apical mobility is stabilized anteriorly without concurrent posterior support, the small bowel may descend caudally into the rectovaginal space, particularly in patients with a deep cul-de-sac or attenuated posterior fascia. These dynamics offer a plausible explanation for early obstructive enterocele after anterior-only sacrocolpopexy, even in the absence of preoperative rectocele or bowel dysfunction. Bowel symptoms were clinically evaluated throughout follow-up and guided diagnostic decisions.

From a surgical standpoint, revision aimed to restore posterior support while preserving the structural integrity of the existing anterior mesh. The posterior mesh was secured to the posterior vaginal wall and anchored to the promontory, allowing functional integration with the previous implant without excessive tension or compartmental distortion.

Earlier recognition of persistent obstructed defecation after anterior-only apical suspension should prompt a low threshold for dynamic imaging, as timely diagnosis may facilitate earlier management and potentially reduce the need for delayed reoperation.

Guidelines and consensus statements reflect these uncertainties. International societies do not mandate systematic placement of both anterior and posterior meshes. Instead, they generally endorse a tailored, compartment-specific approach, reserving posterior mesh for women with symptomatic rectocele or enterocele [19-21]. This individualized strategy balances the risk of posterior recurrence against the potential harms of routine posterior augmentation, including constipation, pelvic pain, dyspareunia, and mesh erosion, which have been reported in 3-13% of cases depending on technique and follow-up [10,22-23].

Future prospective studies, ideally randomized trials, are needed to evaluate whether selective or prophylactic posterior compartment repair provides superior outcomes compared with anterior-only sacrocolpopexy. Specific research questions should include: (a) whether prophylactic posterior repair reduces the incidence of postoperative obstructive enterocele or functional bowel symptoms; (b) whether a selective approach based on intraoperative compartment assessment yields comparable anatomical durability with lower morbidity; and (c) how different strategies impact postoperative bowel function, quality of life, and reoperation rates. Standardized outcomes should include validated bowel function scores, patient-reported functional and quality-of-life measures, anatomical recurrence of posterior compartment defects, postoperative complications, and healthcare utilization.

## Conclusions

This case underscores that while anterior-only sacrocolpopexy is an established and widely used strategy with good outcomes, rare but clinically significant posterior compartment complications, such as obstructive enterocele, may occur. In our patient, symptomatic posterior failure developed early, within six weeks after surgery, emphasizing the need for timely clinical evaluation. Current evidence supports a compartment-specific approach rather than routine dual-compartment mesh placement. Careful patient selection includes confirmation that no symptomatic posterior defect is present, no significant bowel dysfunction exists, and that defecatory complaints have been formally assessed before surgery. Postoperatively, new-onset pelvic pressure, incomplete bowel emptying, rectal tenesmus, or persistent constipation should prompt concern for posterior compartment failure. In such cases, timely imaging, preferably dynamic MRI or defecography, should be obtained to evaluate enterocele or rectocele and guide multidisciplinary management. Future research should explore which perioperative factors may predispose to obstructive enterocele after anterior-only sacrocolpopexy and assess whether individualized postoperative surveillance improves clinical outcomes.
